# Neocolonial echoes in healthcare: ethical quandaries of the Nepal-UK nurse recruitment memorandum of understanding

**DOI:** 10.1016/j.lansea.2024.100493

**Published:** 2024-10-08

**Authors:** Animesh Ghimire, Yunjing Qiu, Mamata Sharma Neupane, Purushottam Ghimire

**Affiliations:** a Sustainable Prosperity Initiative Nepal, Bhimsengola, Thulo Kharibot, Baneshwor-31, Kathmandu, Nepal; b School of Nursing and Midwifery, University of Technology, 15 Broadway, Ultimo NSW 2007, Australia; c School of Nursing, Chitwan Medical College, Bharatpur-5, Nepal; d School of Nursing and Midwifery, Monash University, Wellington Rd, Clayton VIC 3800, Australia

The migration of nurses from the Global South to high income nations in the Global North is more than a mere exchange of labor; it is a story woven with threads of historical power imbalances and colonial legacies. Nepal, long known for its contributions to the UK through the valiant Gurkha regiments,^1^ now faces a new chapter in this narrative. The UK's healthcare system, struggling with severe workforce shortages,^2^ turns to Nepal once again, but this time, it is for its skilled nurses. This shift - from soldiers to nurses-seems driven by necessity on both sides. However, beneath the surface of this mutual need lies a complex web of ethical concerns. Developed nations like the UK, seeking immediate and cost-effective solutions to their own systemic healthcare workforce challenges, often rely on the Global South.^3^ This practice not only exacerbates existing health inequities but also stymies the development of robust healthcare systems in source countries like Nepal. The 2022 Nepal–UK Memorandum of Understanding (MoU),^4^ which began implementation in late 2023,^5^ exemplifies this trend. While it aims to address workforce shortages, it has sparked a heated debate about its ethical implications.

The Nepal–UK MoU is presented as a solution to the UK's pressing healthcare workforce crisis, a crisis exacerbated by an aging population, Brexit-related workforce attrition, and systemic challenges within the National Health Service (NHS) that have hampered recruitment and retention.^6^^,^^7^ However, this agreement starkly contrasts with Nepal's own critical health workforce shortages, as evidenced by its designation on the WHO’s “red list.”^8^ Nepal, with only 3.5 nurses per 1000 people,^9^ is positioned as a key source of nurses for the UK, with 9.2 nurses per 1000 people.^10^ This disparity highlights a troubling power imbalance in the MoU. While the agreement aims to foster ethical and sustainable recruitment through managed migration and reciprocal goals,^4^ some concerns persist it may function as a mere band-aid for the UK's healthcare system rather than a genuine partnership addressing the root causes of global health inequities. This raises crucial ethical questions: Is the UK truly committed to supporting the development of Nepal's healthcare system, or is it merely benefiting from exploiting a vulnerable workforce? Similarly, is Nepal adequately safeguarding its own citizens' right to healthcare amidst this exodus of nurses?

While the Code of Practice for ethical international recruitment prohibits active recruitment from “red-listed” countries like Nepal, a government-to-government agreement excludes this rule.^11^ This allows the UK government to actively recruit nurses from Nepal despite the country's critical health workforce shortages. It is crucial to note that Nepal, classified as a lower-middle-income country in 2020 with a gross national income (GNI) per capita of $1,090,^12^ faces significant challenges in providing adequate healthcare to its population. The exception granted by the MoU, therefore, raises concerns about the potential for exploitation and the exacerbation of existing health inequities. Furthermore, this type of bilateral agreement is not an isolated case, and the UK has established similar MoUs with other countries, including Kenya, Malaysia, the Philippines, India, and Sri Lanka.^11^ Even though Kenya is currently on the “amber list,” active recruitment is still permissible within the confines of their specific MoU.^11^ While the proficiency in the English language of nurses from these countries may facilitate their integration into the NHS, this advantage primarily benefits the UK, enabling them to readily access a skilled and qualified workforce.^13^ This dynamic has been aptly described as “reverse foreign aid” as high-income countries (HICs) essentially benefit from the investment made by developing nations in educating and training their healthcare professionals.^13^

The dissonance between the rhetoric of a globalized “one world” and the reality of nationalistic self-interest during crises exposes the enduring power asymmetries in the global healthcare landscape. HICs, while advocating for a borderless world and global citizenship, often prioritize their own national interests when faced with challenges, revealing a deeply entrenched neo-colonial mindset. This selective prioritization is further amplified by media narratives focusing on staffing crises in HICs, framing them as deviations from an idealized standard of care while overlooking the chronic and systemic struggles of healthcare systems in low and middle-income countries (LMICs). The COVID-19 pandemic starkly exposed this disparity, exemplified by actions like Australia's “airlifting” of nurses, bypassing quarantine requirements for the sake of national interest, further exposing the stark power dynamics at play.^14^

In contrast, LMICs like Nepal, despite having legislation to protect their citizens, often struggle to enforce these protections due to bureaucratic inefficiencies, epistemic injustice, digital coloniality, and significant economic disparities. This struggle is further compounded by the allure of opportunities in high-income countries, which promise better recognition and professional growth, thereby fueling the exodus of healthcare workers. Nepal is already grappling with one of the highest rates of healthcare professional migration in its region^15^; signing this MoU with the UK government raises critical questions about the true motives and beneficiaries of this agreement. If implemented ethically and accompanied by genuine investment in Nepal's healthcare infrastructure and workforce, it could represent a positive step towards a more equitable partnership. However, if it merely serves as a means for the UK to attract skilled nurses from an already resource-constrained country with a struggling healthcare system, it risks perpetuating a cycle of exploitation and global health inequity. For the MoU to truly pass this ethical test, it must demonstrate a commitment to transparency, mutual benefit, and addressing the root causes of nurse migration.

## Contributors

AG—conceptualization and preparation of the first draft.

YQ and PG —critical inputs and revising of the drafts.

YQ, PG, and MSN—finalization and approval of the manuscript.

All authors have read and approved the final version of the manuscript.

## Declaration of interests

We declare no competing interests.
